# Black Ginseng and Ginsenoside Rb1 Promote Browning by Inducing UCP1 Expression in 3T3-L1 and Primary White Adipocytes

**DOI:** 10.3390/nu11112747

**Published:** 2019-11-12

**Authors:** Seon-Joo Park, Miey Park, Anshul Sharma, Kihyun Kim, Hae-Jeung Lee

**Affiliations:** 1 Department of Food and Nutrition, Gachon University, Gyeonggi-do 13120, Korea; chris0825@gachon.ac.kr (S.-J.P.); mieyp@naver.com (M.P.); anshul.silb18@gmail.com (A.S.); 2 Animal Nutrition & Physiology Team, National Institute of Animal Science, Jeolabuk-do 1500, Korea; kihyun@korea.kr

**Keywords:** black ginseng, ginsenoside Rb1, browning effect, UCP1, primary white adipocytes

## Abstract

In this study, we investigated the effects of black ginseng (BG) and ginsenoside Rb1, which induced browning effects in 3T3-L1 and primary white adipocytes (PWATs) isolated from C57BL/6 mice. BG and Rb1 suppressed the expressions of CCAAT/enhancer-binding protein alpha (C/EBPα) and sterol regulatory element-binding transcription factor-1c (SREBP-1c), whereas the expression level of peroxisome proliferator-activated receptor gamma (PPARγ) was increased. Furthermore, BG and Rb1 enhanced the protein expressions of the brown-adipocyte-specific markers PR domain containing 16 (PRDM16), peroxisome proliferator-activated receptor gamma coactivator-1 alpha (PGC-1α), and uncoupling protein 1 (UCP1). These results were further supported by immunofluorescence images of mitochondrial biogenesis. In addition, BG and Rb1 induced expressions of brown-adipocyte-specific marker proteins by AMP-activated protein kinase (AMPK) activation. BG and Rb1 exert antiobesity effects by inducing browning in 3T3-L1 cells and PWATs through AMPK-mediated pathway activation. We suggest that BG and Rb1 act as potential functional antiobesity food agents.

## 1. Introduction

Obesity is a metabolic disorder characterized by hyperplasia and hypertrophy of white adipose tissue (WAT) and is associated with several comorbidities, including cardiovascular disease, hypertension, stroke, diabetes, cancer, and nonalcoholic fatty liver disease [1,2]. The important features of WAT include storage of excess energy as triglycerides and release of adiponectin and leptin for energy homeostasis [3,4]. In contrast, brown adipose tissue (BAT) utilizes energy as heat through the oxidation of fatty acids. There are two types of BAT in mammals: classical BAT and brown-like adipose tissue. Brown-like adipose tissue is present within WAT and undergoes a transition to brown-in-white tissue after induction [1,5,6]. Brown-like adipose tissue is also known as brite, beige, or induced and its structural and functional aspects are similar to classical BAT [1]. The discovery of BAT has opened new options for formulating novel strategies to combat obesity and associated risk factors [7]. Therefore, besides suggesting dietary restrictions and physical activity as remedies for diet-induced obesity, browning induction has been considered an alternative strategy [3].

The leading functional factor of BAT is uncoupling protein 1 (UCP1), which is involved in adaptive thermogenesis [8,9]. UCP1 is localized in the inner mitochondrial membrane of BAT, generating heat instead of ATP by disturbing the proton gradient of the membrane [10]. Furthermore, the other hallmark proteins of BAT include a PR domain containing 16 (PRDM16) and peroxisome proliferator-activated receptor gamma (PPARγ) coactivator-1 alpha (PGC-1α). To date, the thermogenic potential of bioactive agents has been demonstrated by many studies, involving the transition of WAT to beige adipose tissue types [11,12,13,14].

Ginseng (*Panax ginseng* C.A. Meyer) has been a popular traditional herbal supplement in the Korean Peninsula for centuries. Black ginseng (BG) is processed by drying and steaming ginseng nine times. The steaming process results in the conversion of ginsenosides of white and red ginseng into less polar types, and 19 ginsenosides (Rb1, Rb2, Rc, Rd, Re, Rf, Rg1, Rg6, F4, Rk3, Rh4, 20(S)-, 20(R)-Rg3, 20(S)-, 20(R)-Rs3, Rk1, Rg5, Rs4, and Rs5) have been newly discovered in BG [15]. BG has much more substantial biological activities than white and red ginseng, including antiobesity, antidiabetes, anticancer, anti-inflammatory, antinociceptive, antioxidant, antihyperglycemic, and immune-modulating activities [16,17,18,19,20]. In addition, the health-promoting activities of ginsenoside Rb1 have also been documented [21]. For example, Rb1 has been reported to exhibit antidiabetic potential by improving glucose tolerance [22,23], as well as antiobesity [22], anti-inflammatory, immunomodulatory [24,25], hepatoprotective [26], antiatherosclerotic [21], and neuroprotective [27] bioactivities. Furthermore, Shang et al. demonstrated the protective effects of Rb1 against adipogenesis by increasing expressions of PPARγ and CCAAT/enhancer-binding protein alpha (C/EBPα) [28]. Consistent with the study by Shang et al., recent studies have described the antiobesity effects of ginseng and ginsenosides, including the induction of browning by Rg1 in 3T3-L1 cells and subcutaneous WAT [2,3,29]. These findings suggest that ginsenosides from ginseng play an important role as bioactive components. However, little information is available on the bioactivities of Korean BG and saponins associated with browning and obesity. Thus, the present study aimed to examine the antiobesity activity of BG and ginsenoside Rb1 in 3T3-L1 preadipocytes and primary white adipocytes (PWATs) via induction of the browning mechanism. To our knowledge, this is the first investigation into the browning effects of BG and Rb1 on 3T3-L1 cells and PWATs.

## 2. Materials and Methods

### 2.1. Materials

The u-HPLC analysis of BG showed that the total ginsenoside content in the roots and leaves were 21.00 and 22.94 mg/g, respectively (JGR, Park et al., 2019 in press) [30].

### 2.2. Cell Culture and Differentiation

The 3T3-L1 preadipocytes were obtained from the American Type Culture Collection (ATCC, Manassas, VA, USA) and were maintained in Dulbecco’s Modified Eagle’s Medium (DMEM) supplemented with 10% bovine calf serum (BCS) and 1% antibiotics. The cells were grown at 37 °C in a CO_2_ (5%) incubator. After the cells reached confluence, differentiation of 3T3-L1 cells was initiated by incubating the cells in differentiation induction medium (0.5 mΜ methylisobutylxanthine (IBMX), 1 μΜ dexamethasone (DEX), and 5 μg/mL of insulin) in DMEM including 10% fetal bovine serum (FBS). After 3 days, the differentiation medium was replaced with postdifferentiation medium consisting of 10% FBS and 5 μg/mL of insulin. The medium was changed every 2 days, which was continued until the 3T3-L1 preadipocytes were fully differentiated into white adipocytes. On day 7, the fully differentiated white adipocytes were used for further experiments.

### 2.3. Primary White Adipocyte Preparation

Subcutaneous fat tissues of 5–6-week-old male C57BL/6 mice were used for the isolation of PWATs. Briefly, subcutaneous tissues were crushed with scissors and digested with collagenase type II enzyme (Sigma-Aldrich, St. Louis, MO, USA) in a water bath at 37 °C for 1 h. The mixture was filtered using 40 μm cell strainers (SPL Life Science, Gyeonggi, Korea) and centrifuged at 300× *g* for 7 min. The pellet consisting of stromal vascular fractions was resuspended in DMEM supplemented with 10% BCS and 1% antibiotics. After reaching confluence, differentiation was initiated by incubating the cells for 3 days in the differentiation induction medium (the composition was the same as described in Section 2.2). The animal tissue experiments followed the Guidelines for the Care and Use of Laboratory Animals of Gachon University (reference number: GIACUC-R2018016).

### 2.4. BG and Rb1 Treatment

To evaluate the browning effects, 3T3-L1 preadipocytes and PWATs were incubated with BG (25, 50, and 100 μg/mL) and Rb1 (10, 20, and 40 μM) from day 0 to 7. Differentiated cells without supplements of BG and Rb1 were used as controls.

### 2.5. Cell Viability Assay

The cytotoxicity of BG and Rb1 was measured using a Cell Counting Kit-8 (CCK-8) (Dojindo Molecular Technologies, Rockville, MD, USA) according to the manufacturer’s instructions. 3T3-L1 preadipocytes were seeded in a 96-well plate at a density of 5 × 10^4^ cells/well and treated with varying concentrations of BG and Rb1 for 24, 48, and 72 h. Finally, the cells were treated with 10 μL of CCK-8 and incubated at 37 °C for 2 h. The absorbance was measured at 450 nm using a microplate spectrophotometer system (BioRad, Hercules, CA, USA). The results were expressed as a percentage of cell viability and each experiment was repeated three times.

### 2.6. Oil Red O Staining

Lipid droplets in the differentiated 3T3-L1 adipocytes were detected by Oil Red O staining. Differentiated adipocytes were washed with phosphate-buffered saline (PBS), fixed with 10% formalin (Thermo Fisher Scientific, Waltham, MA, USA), and dried working solution (60% isopropanol and 40% distilled water, *w*/*v*) was added to each well at room temperature (RT) for 30 min. The cells were washed three times with deionized water and observed under an inverted microscope. Finally, the dye was eluted using 100% isopropanol to quantify the intracellular lipid content, and the absorbance was determined at 500 nm.

### 2.7. Immunoblot Analysis 

Cell lysates were prepared using protein lysis buffer with protease inhibitor cocktail (Thermo Fisher Scientific, Waltham, MA, USA). The lysates were diluted in 5× sample buffer and heated at 95 °C for 5 min, and equal amounts of protein (30 μg) from each sample were separated by 10% sodium dodecyl sulfate-polyacrylamide gel electrophoresis (SDS-PAGE). The SDS-PAGE proteins were electrotransferred onto polyvinylidene difluoride (PVDF) membranes (Merck Millipore, Burlington, MA, USA). The membrane was blocked with 5% skim milk at RT for 1 h under shaking conditions. The membranes were washed three times with 1× PBS with Tween 20 (PBST), followed by incubation with primary antibodies, which included UCP1, PRDM16, PGC-1α, PPARγ, C/EBPα (1:1000, Abcam, Cambridge, MA, USA), sterol regulatory element-binding transcription factor-1c (SREBP-1c, 1:1000, Santa Cruz Biotechnology, Dallas, CA, USA), AMP-activated protein kinase (AMPK), AMPK phosphorylation (*p*-AMPK) (1:1000, Cell Signaling Technology, Danvers, MA, USA), and β-actin (1:5000, Abcam) at 4 °C overnight. After washing, the membranes were incubated with horseradish peroxidase (HRP)-coupled secondary antibodies (1:1000) at RT for 1 h. The band signals were detected using an ECL Western blot detection kit (Amersham Pharmacia, Little Chalfont, Bucks, UK) and Image Quant LAS 500 (GE Healthcare Bio-Sciences AB, Uppsala, Sweden) and expressed as a percentage of the change relative to the expression of β-actin. The *p*-AMPK levels were normalized to the total AMPK levels.

### 2.8. Effect of BG and Rb1 on Adipogenesis and Browning Markers

To evaluate the effect of BG and Rb1 on adipogenesis, the expression of adipogenic marker proteins, including PPARγ, C/EBPα, and SREBP-1c, were examined by Western blot analysis in both 3T3-L1 cells and PWATs. Furthermore, to evaluate browning, the expression of BAT-specific marker proteins (UCP1, PRDM16, and PGC-1α) was investigated by immunoblotting in both models with and without treatment with varying concentrations of BG and Rb1. Each experiment was repeated three times.

### 2.9. Immunofluorescence Staining

Immunofluorescence was used to evaluate the effects of BG and Rb1 on fundamental protein, UCP1, and mitochondrial biogenesis using both 3T3-L1 cells and PWATs. For immunocytochemical analyses, differentiated adipocytes were fixed with 4% formaldehyde and permeabilized with 0.2% Triton X-100 (Sigma, St. Louis, MO, USA), followed by blocking with 1% BSA in PBST for 30 min. After that, the cells were incubated with anti-UCP1 antibody (1:250, Abcam) overnight at 4 °C. The cells were washed with PBS and incubated with fluorescein isothiocyanate (FITC)-conjugated anti-rabbit secondary antibody (1:500, Promega, Madison, WI, USA) in 1% BSA for 1 h. Mitochondria were stained using MitoTracker Red CMXRos (500 nM; Cell Signaling Technology) for 30 min according to the manufacturer’s protocol. Then, the cells were fixed and rinsed three times with PBS. The nuclei of the cells were stained with 4′,6-diamidino-2-phenylindole (DAPI; Invitrogen, Carlsbad, MA, USA). Fluorescence images were captured by using an inverted phase-contrast microscope with fluorescence (KI-2000F, Korea Lab Tech, Gyeonggi, Korea) and analyzed with image-processing software (OptiView 3.7, Korea Lab Tech, Gyeonggi, Korea).

### 2.10. Induction of Browning by AMPK Activation

3T3-L1 and PWAT cells were treated with an AMPK activator (5-aminoimidazole-4-carboxamide ribonucleotide (AICAR)) and an AMPK inhibitor (dorsomorphin; Sigma-Aldrich, St. Louis, MO, USA) to examine the browning effects caused by BG and Rb1. AICAR (10 μΜ) or dorsomorphin (5 μΜ) was added to the differentiation induction and maturation media until the cells were harvested. Each experiment was repeated three times. To further evaluate the possible mechanism of browning, we measured the expression of *p*-AMPK and browning hallmark proteins (UCP1, PRDM16, and PGC-1α) in the presence of AICAR and dorsomorphin in PWATs.

### 2.11. Statistical Analyses

All data are expressed as mean ± standard deviation (SD). All experiments were performed in triplicate. One-way ANOVA and Tukey’s post hoc tests were performed using GraphPad Prism 5.03 (GraphPad Software Inc., San Diego, CA, USA). *p*-values less than 0.05 indicted statistical significance.

## 3. Results

### 3.1. Effect of BG and Rb1 on the Differentiation in 3T3-L1 Cells

**Figure 1:**
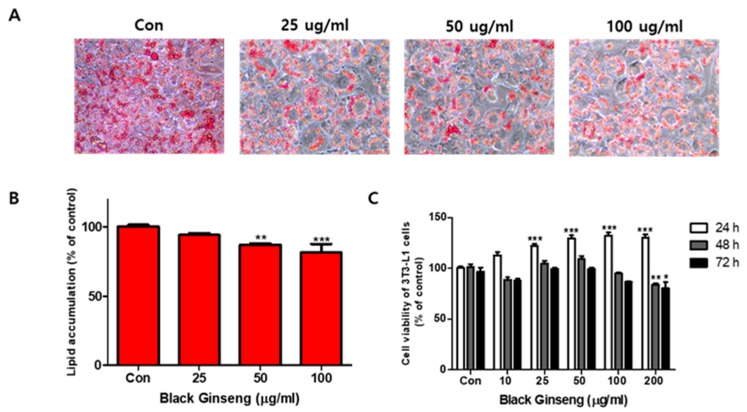
Effect of black ginseng (BG) on lipid accumulation and cell viability in 3T3-L1 adipocytes. (**A**) Oil Red O staining was used to visualize the lipid droplets to evaluate the differentiation of 3T3-L1 cells in the presence (25, 50, and 100 µg/mL) or absence of BG (control; Con). (**B**) Lipid accumulation was quantified. (**C**) Relative viability of 3T3-L1 cells in the presence of BG, evaluated using a CCK-8 assay. The data are presented as mean ± SD for three different experiments. * *p* < 0.05, ** *p* < 0.01, and *** *p* < 0.001 vs. Con.

**Figure 2:**
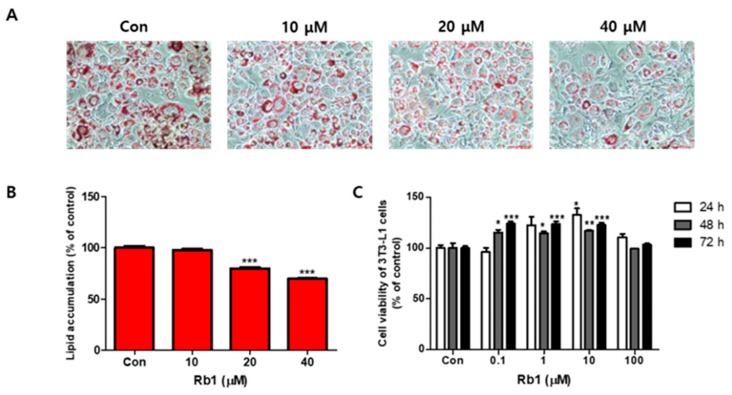
Effect of ginsenoside Rb1 on lipid accumulation and cell viability in 3T3-L1 adipocytes. (**A**) Oil Red O staining was used to visualize the lipid droplets to evaluate the differentiation of 3T3-L1 cells in the presence (10, 20, and 40 µM) or absence of Rb1 (Con). (**B**) Lipid accumulation was quantified. (**C**) Relative viability of 3T3-L1 cells in the presence of Rb1, evaluated using a CCK-8 assay. The data are presented as mean ± SD for three different experiments. * *p* < 0.05, ** *p* < 0.01 and *** *p* < 0.001 vs. Con.

The effects of BG and Rb1 on 3T3-L1 cell differentiation are shown in Figure 1 and Figure 2, respectively. Fully differentiated 3T3-L1adipocytes not treated with BG or Rb1 showed typical lipid accumulation by Oil Red O staining. The lipid droplets were decreased by treatment with BG (Figure 1A) and Rb1 (Figure 2A) and lipid accumulation in the differentiated adipocytes was significantly reduced upon treatment with BG (50 and 100 μg/mL; Figure 1B) and Rb1 (20 and 40 μΜ; Figure 2B). The CCK-8 assay results revealed that BG and Rb1 had no significant cytotoxicity at 100 μg/mL BG or Rb1concentrations below 100 μΜ (Figure 1C and Figure 2C, respectively).

### 3.2. Effect of BG and Rb1 on Adipogenesis in 3T3-L1 and Primary White Adipocytes

**Figure 3:**
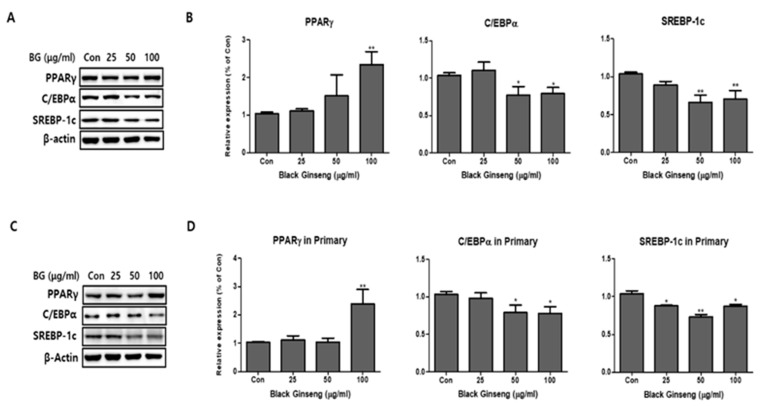
Effect of BG on adipogenesis in 3T3-L1 and primary white adipocytes (PWATs). Protein expressions of peroxisome proliferator-activated receptor gamma (PPARγ), CCAAT/enhancer-binding protein alpha (C/EBPα), and sterol regulatory element-binding transcription factor-1c (SREBP-1c) were measured by Western blotting at different concentrations of BG (25, 50, and 100 µg/mL) in both 3T3-L1 (**A**,**B**) and PWATs (**C**,**D**) or absence of BG (Con). The β-actin protein was used as an internal control. The data are presented as mean ± SD for three different experiments. * *p* < 0.05 and ** *p* < 0.01 vs. Con.

**Figure 4:**
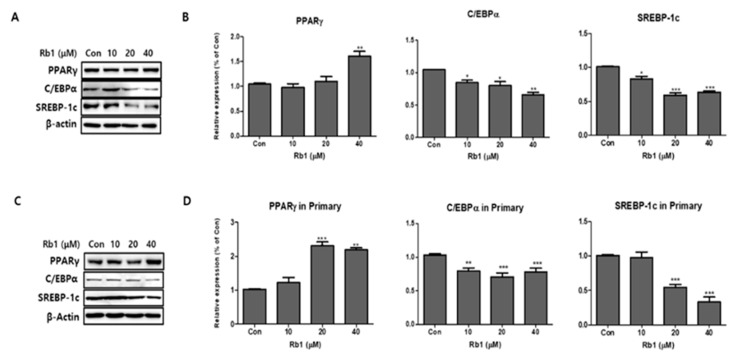
Effect of ginsenoside Rb1 on adipogenesis in 3T3-L1 and PWATs. Protein expressions of PPARγ, C/EBPα, and SREBP-1c were measured by Western blotting at different concentrations of Rb1 (10, 20, and 40 μM) in both 3T3-L1 (**A**,**B**) and PWATs (**C**,**D**) or absence of Rb1 (Con). The β-actin protein was used as an internal control. The data are presented as mean ± SD for three different experiments. * *p* < 0.05, ** *p* < 0.01, and *** *p* < 0.001 vs. Con.

We added BG and Rb1 during the differentiation of 3T3-L1 cells and PWATs. The adipogenic markers PPARγ, C/EBPα, and SREBP-1c were examined by Western blot analysis. After differentiation, BG increased the expression levels of PPARγ in 3T3-L1 cells in both types of adipocytes in a dose-dependent manner (Figure 3). Notably, 100 μg/mL of BG significantly increased the expression of PPARγ compared with the control group. The relative Western blot expressions of C/EBPα and SREBP-1c were inhibited by BG treatment in dose-dependent manners in both 3T3-L1 cells and PWATs. As shown in Figure 4, Rb1 treatment upregulated the expression of PPARγ in both types of adipocytes. However, the increase was not observed in a dose-dependent manner in PWATs. In contrast, Rb1 substantially inhibited C/EBPα and SREBP-1c expression in a dose-dependent manner in both 3T3-L1 cells and PWATs.

### 3.3. Effect of BG and Rb1 on the Expression of Brown Adipocyte Markers in 3T3-L1 Cells and PWATs

**Figure 5:**
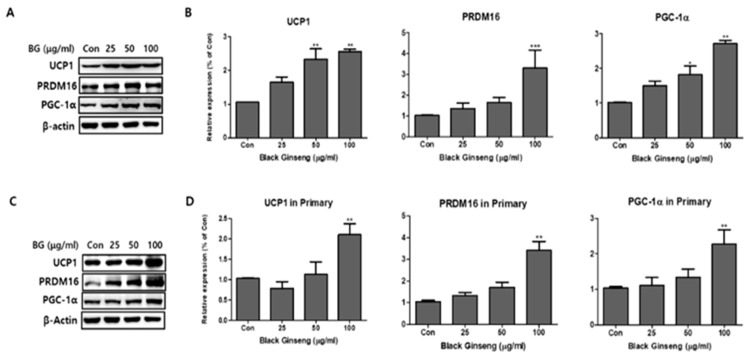
Effect of BG on expression of brown adipocyte markers. Protein expressions of uncoupling protein 1 (UCP1), PR domain containing 16 (PRDM16), and PPARγ coactivator-1 alpha (PGC-1α) were measured by Western blotting at different concentrations of BG (25, 50, and 100 µg/mL) in both 3T3-L1 (**A**,**B**) and PWATs (**C**,**D**) or absence of BG (Con). The β-actin protein was used as an internal control. The data are presented as mean ± SD for three different experiments. * *p* < 0.05, ** *p* < 0.01, and *** *p* < 0.001 vs. Con.

**Figure 6:**
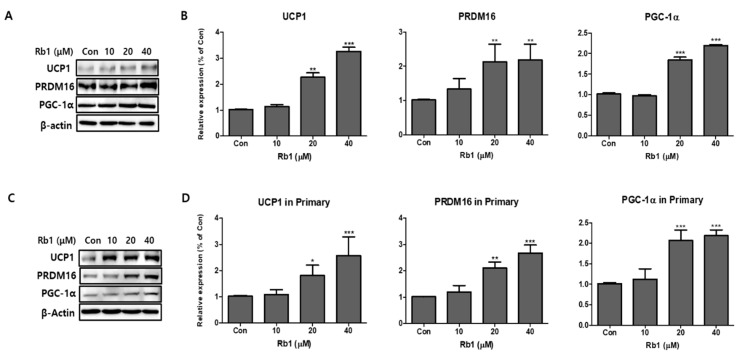
Effect of ginsenoside Rb1 on expression of brown adipocyte markers. Protein expressions of UCP1, PRDM16, and PGC-1α were measured by Western blotting at different concentrations of Rb1 (10, 20, and 40 µM) in both 3T3-L1 (**A**,**B**) and PWATs (**C**,**D**) or absence of Rb1 (Con). The β-actin protein was used as an internal control. The data are presented as mean ± SD for three different experiments. * *p* < 0.05, ** *p* < 0.01, and *** *p* < 0.001 vs. Con.

To investigate the browning effects of BG and Rb1, 3T3-L1 cells and PWATs were treated with BG and Rb1. Western blot analysis revealed that 100 μg/mL BG treatment significantly increased the expression of brown adipocyte markers (UCP1, PRDM16, and PGC-1α) in both types of adipocytes (Figure 5). In addition, UCP1 and PGC-1α were upregulated in 3T3-L1 adipocytes treated with 50 μg/mL of BG. Rb1 treatments (20 and 40 μΜ) also significantly increased the expression of the brown adipocyte markers in both types of adipocytes in dose-dependent manners (Figure 6).

### 3.4. Immunofluorescence

**Figure 7:**
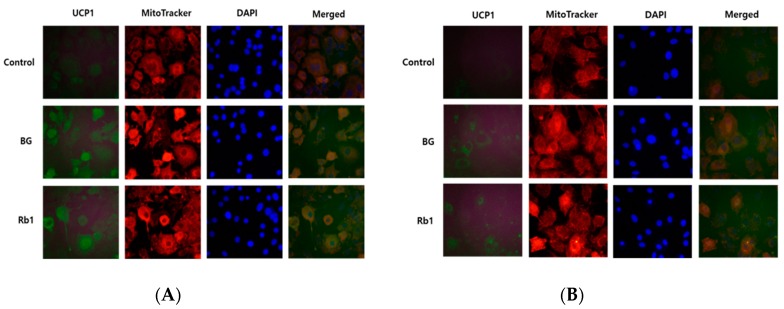
Effects of BG and Rb1 treatment on UCP1 protein expression in 3T3-L1 cells and PWATs. 3T3-L1 cells and PWATs treated with BG (**A**) and Rb1 (**B**) were subjected to immunofluorescence for UCP1 and stained with MitoTracker Red and 4′,6-diamidino-2-phenylindole (DAPI). The immunofluorescent images were captured at 200× magnification.

As described above, BG and Rb1 treatments resulted in increased expression of BAT-specific marker proteins (UCP1, PRDM16, and PGC-1α), which suggests the possibility of browning in 3T3-L1 cells and PWATs. Immunofluorescence was used to evaluate the effects of BG and Rb1 on fundamental protein, UCP1, and mitochondrial biogenesis. Differentiated 3T3-L1 cells and PWATs were stained with MitoTracker Red (red), then incubated with UCP1 antibody (green). As shown in Figure 7A,B, mitochondrial density was significantly increased in both BG- and Rb1-treated adipocytes, which was confirmed by enhanced red fluorescent dye binding to mitochondria. Likewise, the expression of UCP1 (green) was enhanced in the 3T3-L1 cells and PWATs by BG and Rb1 treatment.

### 3.5. Effect of BG and Rb1 on Activation of AMPK

**Figure 8:**
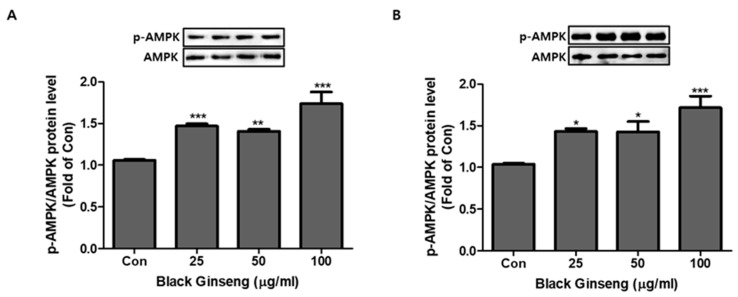
Effect of BG on activation of AMP-activated protein kinase (AMPK). Dose-dependent effect of BG on expression of AMPK phosphorylation (*p*-AMPK)/AMPK in 3T3-L1 cells (**A**) and PWATs (**B**). Expressions were measured by Western blotting. The data are presented as mean ± SD for three different experiments. * *p* < 0.05, ** *p* < 0.01, and *** *p* < 0.001 vs. Con.

**Figure 9:**
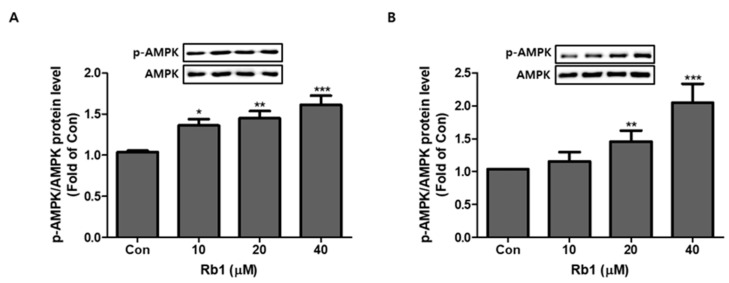
Effect of Rb1 on activation of AMPK. Dose-dependent effect of Rb1 on expression of *p*-AMPK/AMPK in 3T3-L1 cells (**A**) and PWATs (**B**). Expressions were measured by Western blotting. The data are presented as mean ± SD for three different experiments. * *p* < 0.05, ** *p* < 0.01, and *** *p* < 0.001 vs. Con.

To determine the possible mechanism of adipocyte browning by BG and Rb1, we measured the expression levels of AMPK and *p*-AMPK. The ratios of *p*-AMPK/AMPK were significantly increased following BG and Rb1 treatment of 3T3-L1 cells (Figure 8A and Figure 9A) and PWATs (Figure 8B and Figure 9B).

### 3.6. BG and Rb1 Induces Browning Effect via AMPK-Mediated Pathway

**Figure 10:**
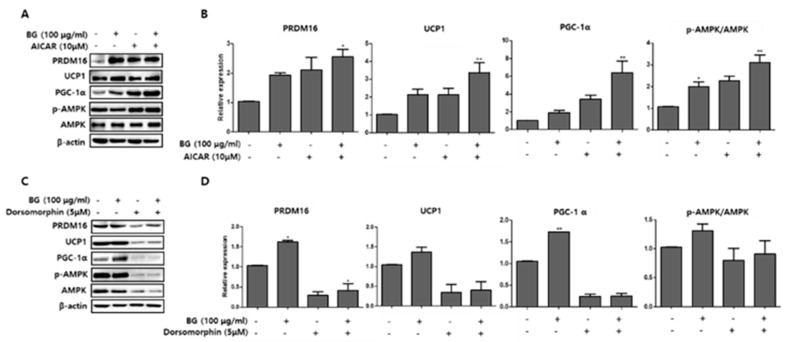
Effect of 5-aminoimidazole-4-carboxamide ribonucleotide (AICAR) and dorsomorphin in the presence of BG on the expression of brown adipocyte markers. AICAR (**A**) and dorsomorphin (**B**) were added to PWATs. Protein expression levels of PRDM16, UCP1, PGC-1α, and *p*-AMPK/AMPK were evaluated by Western blotting. The data are presented as mean ± SD for three different experiments. * *p* < 0.05 and ** *p* < 0.01 vs. Con.

**Figure 11:**
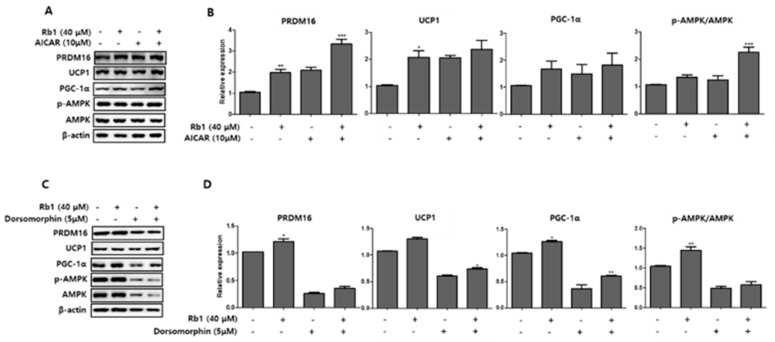
Effect of AICAR and dorsomorphin in the presence of Rb1 on the expression of brown adipocyte markers. AICAR (**A**) and dorsomorphin (**B**) were added to PWATs. Protein expression levels of PRDM16, UCP1, PGC-1α, and *p*-AMPK/AMPK were evaluated by Western blotting. The data are presented as mean ± SD for three different experiments. * *p* < 0.05, ** *p* < 0.01, and *** *p* < 0.001 vs. Con.

To further examine the mechanism of the browning effects of BG and Rb1, we measured the expression of AMPK phosphorylation and UCP1, PRDM16, and PGC-1α browning hallmark proteins following treatment with an AMPK activator (AICAR; 10 µM) and an AMPK inhibitor (dorsomorphin; 5 µM) in PWATs. As shown in Figure 10, BG treatment in the presence of AICAR increased the expression of UCP1, PRDM16, and PGC-1α and increased the *p*-AMPK/AMPK expression ratio, while dorsomorphin (inhibitor) abolished the expressions of these proteins compared with the control. Similarly, Rb1 treatment also increased the *p*-AMPK/AMPK expression ratio and the browning markers compared with controls in PWAT cells (Figure 11).

## 4. Discussion

The prevalence of obesity and associated metabolic disorders continues to increase worldwide. Since browning leads to weight loss through energy expenditure, research related to increasing thermogenesis or browning has gained momentum recently. Therefore, the use of recruitable brown adipocytes represents a promising strategy to treat and prevent obesity [31]. For this reason, functional foods or bioactive ingredients are being explored for inducing browning using in vivo and in vitro models.

In the present study, the effects of BG and Rb1 in 3T3-L1 cells and PWATs were investigated to determine whether the characteristics of white adipocytes could be converted to those of brown-like adipocytes. The browning effects were evaluated by measuring the expression of PRDM16, UCP1, and PGC-1 brown-adipocyte-specific hallmark proteins in both types of adipocytes. Our results show that BG and Rb1 significantly upregulated the expression of PRDM16, UCP1, and PGC-1 in dose-dependent manners in both models. Moreover, mechanistic implications suggest that the target compounds used in the present study activated the AMPK signaling cascade responsible for the increased expression of hallmark proteins, thereby promoting browning in 3T3-L1 cells and PWATs.

UCP1 is a hallmark brown adipocyte protein that plays an important role in thermogenesis by uncoupling electron transport from ATP [10]. In this study, increased expression of UCP1 showed that mitochondrial activity was essential for the browning effect. Our results were further supported by immunofluorescence assays, which showed that BG and Rb1 increased UCP1 expression and mitochondrial activity. An increase in the number of mitochondria is an important feature of beige adipocytes [1]. Consistent with our findings, Lee et al. demonstrated that ginsenoside Rg1 induced browning by increasing UCP1 expression and mitochondrial activity in 3T3-L1 cells and subcutaneous WAT. According to the authors, the antiobesity effects of Rg1 were mediated through AMPK activation [3].

PGC-1α is an important modulator of mitochondrial biogenesis and function and its expression in WAT drives the development of BAT-like characteristics. We demonstrated that BG and Rb1 increased the expression of PGC-1α in 3T3-L1 cells and PWATs in dose-dependent manners, which suggests that the target compounds accelerated energy expenditure. In addition, PGC-1α activation has been reported to induce the expression of essential energy hemostasis proteins, namely, UCP1 and PRDM16. PRDM16 is a coregulatory transcriptional marker and a key switch in the development of BAT and brown-like adipose tissue in WAT [32]. In this study, PRDM16 expression was upregulated by BG and Rb1 treatments. According to Wang and Seale, transcriptional factors, including PRDM16, which are expressed on brown and beige cells, cooperate with other adipogenic transcriptional factors, such as PPARγ, to drive browning and thermogenic activities [33]. PPARγ is a key regulator in the coordination of various molecular actions that confirm the normal physiological function of white, brown, and beige adipocytes. Formation of a functional transcriptional complex (PPARγ/PRDM16/PGC-1α) is believed to be involved in the regulation of brown and beige-like characteristics [34]. Our results showed that BG and Rb1 significantly increased the expression of PPARγ in both adipocyte models. Mu et al. demonstrated that Rb1 promoted browning in 3T3-L1 cells by activating PPARγ, a result that was supported by increased mRNA expressions of PRDM16, UCP1, and PGC-1α [35].

Furthermore, the process of transdifferentiation is further promoted by downregulating the protein expression of lipogenic transcriptional factors, including C/EBPα and SREBP-1c. The reduced size and accumulation of lipids in the form of small lipid droplets observed after Oil Red O staining provide further evidence. These findings imply that BG and Rb1 treatments resulted in reduced expressions of C/EBPα and SREBP1-c.

AMPK is a well-known regulator of metabolism, and activation of the AMPK-mediated pathway is associated with cellular energy homeostasis sensing [36,37]. AMPK plays an important role in the activation of UCP1, a key protein in the browning event [38]. Our data showed that the target compounds significantly upregulated the AMPK expression, along with its phosphorylated form (activated) in both models. Furthermore, a direct interaction between PGC-1α and AMPK has also been reported to be essential for thermogenic activity [39]. Besides increasing mitochondrial number and the expression of brown-specific marker proteins, BG and Rb1 significantly increased the relative expressions of *p*-AMPK and AMPK.

To further evaluate the roles of BG and Rb1 in AMPK activation, PWATs isolated from C57BL/6 mice were treated with AICAR and dorsomorphin in the presence of BG and Rb1. As shown in Figure 10 and Figure 11, both target compounds enhanced the phosphorylation of AMPK and the relative expressions of PRDM16, UCP1, and PGC-1α in the presence of AICAR, while the expressions were abolished in the presence of dorsomorphin. Thus, our data demonstrated that both BG and Rb1 played a crucial role in the activation of the AMPK signaling cascade, which subsequently led to the enhanced expression of brown-specific marker proteins. Consistently, many studies have reported the browning effects of natural bioactive compounds by activation of the AMPK-mediated pathway [13,14,40]. Hence, we suggest that BG and Rb1 treatments might stimulate brown-like adipose tissue in PWATs by upregulating the expression of hallmark proteins, including PRDM16, UCP1, and PGC-1α. Overall, our data suggest that BG and Rb1 promoted browning by inducing the expression of brown-specific hallmark proteins through AMPK activation in 3T3-L1 cells and PWATs. These results are the first to report the browning effects of BG and Rb1 using in vitro and ex vivo models.

We elucidated the role of BG and Rb1 in the browning of 3T3-L1 cells and PWATs. The browning effects were mediated by increasing expressions of UCP1, PRDM16, PGC-1, and PPARγ, as well as decreased expressions of C/EBPα and SREBP1, inhibition of lipid accumulation, and activation of the AMPK signaling cascade. Therefore, the data suggest that BG and Rb1 might be potential therapeutic candidates for the prevention and treatment of obesity.

## References

[1] Harms M., Seale P. (2013). Brown and beige fat: Development, function and therapeutic potential. Nat. Med..

[2] Zhang L., Virgous C., Si H. (2017). Ginseng and obesity: Observations and understanding in cultured cells, animals and humans. J. Nutr. Biochem..

[3] Lee K., Seo Y.-J., Song J.-H., Lee B.-Y. (2018). Ginsenoside Rg1 promotes browning by inducing UCP1 expression and mitochondrial activity in 3T3-L1 and subcutaneous white adipocytes. J. Ginseng Res..

[4] Wang S., Pan M.-H., Hung W.-L., Tung Y.-C., Ho C.-T. (2019). From White to Beige Adipocytes: Therapeutic Potential of Dietary Molecules Against Obesity and Their Molecular Mechanisms. Food Funct..

[5] Wu J., Boström P., Sparks L.M., Ye L., Choi J.H., Giang A.-H., Khandekar M., Virtanen K.A., Nuutila P., Schaart G. (2012). Beige adipocytes are a distinct type of thermogenic fat cell in mouse and human. Cell.

[6] Nedergaard J., Cannon B. (2014). The browning of white adipose tissue: Some burning issues. Cell Metab..

[7] Srivastava S., Veech R.L. (2019). Brown and Brite: The Fat Soldiers in the Anti-obesity Fight. Front. Physiol..

[8] Elsen M., Raschke S., Tennagels N., Schwahn U., Jelenik T., Roden M., Romacho T., Eckel J. (2013). BMP4 and BMP7 induce the white-to-brown transition of primary human adipose stem cells. Am. J. Physiol. Cell Physiol..

[9] Tseng Y.-H., Kokkotou E., Schulz T.J., Huang T.L., Winnay J.N., Taniguchi C.M., Tran T.T., Suzuki R., Espinoza D.O., Yamamoto Y. (2008). New role of bone morphogenetic protein 7 in brown adipogenesis and energy expenditure. Nature.

[10] Sidossis L., Kajimura S. (2015). Brown and beige fat in humans: Thermogenic adipocytes that control energy and glucose homeostasis. J. Clin. Investig..

[11] Leu S.-Y., Tsai Y.-C., Chen W.-C., Hsu C.-H., Lee Y.-M., Cheng P.-Y. (2018). Raspberry ketone induces brown-like adipocyte formation through suppression of autophagy in adipocytes and adipose tissue. J. Nutr. Biochem..

[12] Kang N.H., Mukherjee S., Yun J.W. (2019). Trans-Cinnamic Acid Stimulates White Fat Browning and Activates Brown Adipocytes. Nutrients.

[13] Lone J., Choi J.H., Kim S.W., Yun J.W. (2016). Curcumin induces brown fat-like phenotype in 3T3-L1 and primary white adipocytes. J. Nutr. Biochem..

[14] Zhang Z., Zhang H., Li B., Meng X., Wang J., Zhang Y., Yao S., Ma Q., Jin L., Yang J. (2014). Berberine activates thermogenesis in white and brown adipose tissue. Nat. Commun..

[15] Sun B.-S., Gu L.-J., Fang Z.-M., Wang C.-Y., Wang Z., Lee M.-R., Li Z., Li J.-J., Sung C.-K. (2009). Simultaneous quantification of 19 ginsenosides in black ginseng developed from Panax ginseng by HPLC–ELSD. J. Pharm. Biomed. Anal..

[16] Kang O.-H., Shon M.-Y., Kong R., Seo Y.-S., Zhou T., Kim D.-Y., Kim Y.-S., Kwon D.-Y. (2017). Anti-diabetic effect of black ginseng extract by augmentation of AMPK protein activity and upregulation of GLUT2 and GLUT4 expression in db/db mice. BMC Complement. Altern. Med..

[17] Lee M.R., Ma J.Y., Sung C.K. (2017). Chronic dietary ginseng extract administration ameliorates antioxidant and cholinergic systems in the brains of aged mice. J. Ginseng Res..

[18] Kim S.-J., Kim A.K. (2015). Anti-breast cancer activity of Fine Black ginseng (Panax ginseng Meyer) and ginsenoside Rg5. J. Ginseng Res..

[19] Seo Y.S., Shon M.Y., Kong R., Kang O.H., Zhou T., Kim D.Y., Kwon D.Y. (2016). Black ginseng extract exerts anti-hyperglycemic effect via modulation of glucose metabolism in liver and muscle. J. Ethnopharmacol..

[20] Lee Y.Y., Saba E., Irfan M., Kim M., Chan J.Y.-L., Jeon B.S., Choi S.K., Rhee M.H. (2019). The anti-inflammatory and anti-nociceptive effects of Korean black ginseng. Phytomedicine.

[21] Zhou P., Lu S., Luo Y., Wang S., Yang K., Zhai Y., Sun G., Sun X. (2017). Attenuation of TNF-α-induced inflammatory injury in endothelial cells by ginsenoside Rb1 via inhibiting NF-κB, JNK and p38 signaling pathways. Front. Pharmacol..

[22] Xiong Y., Shen L., Liu K.J., Tso P., Xiong Y., Wang G., Woods S.C., Liu M. (2010). Antiobesity and antihyperglycemic effects of ginsenoside Rb1 in rats. Diabetes.

[23] Zhou P., Xie W., He S., Sun Y., Meng X., Sun G., Sun X. (2019). Ginsenoside Rb1 as an Anti-Diabetic Agent and Its Underlying Mechanism Analysis. Cells.

[24] Zhang X.-J., He C., Tian K., Li P., Su H., Wan J.-B. (2015). Ginsenoside Rb1 attenuates angiotensin II-induced abdominal aortic aneurysm through inactivation of the JNK and p38 signaling pathways. Vasc. Pharmacol..

[25] Yu J., Eto M., Akishita M., Kaneko A., Ouchi Y., Okabe T. (2007). Signaling pathway of nitric oxide production induced by ginsenoside Rb1 in human aortic endothelial cells: A possible involvement of androgen receptor. Biochem. Biophys. Res. Commun..

[26] Yu X., Ye L., Zhang H., Zhao J., Wang G., Guo C., Shang W. (2015). Ginsenoside Rb1 ameliorates liver fat accumulation by upregulating perilipin expression in adipose tissue of db/db obese mice. J. Ginseng Res..

[27] Hwang J.Y., Shim J.S., Song M.-Y., Yim S.-V., Lee S.E., Park K.-S. (2016). Proteomic analysis reveals that the protective effects of ginsenoside Rb1 are associated with the actin cytoskeleton in β-amyloid-treated neuronal cells. J. Ginseng Res..

[28] Shang W., Yang Y., Jiang B., Jin H., Zhou L., Liu S., Chen M. (2007). Ginsenoside Rb1 promotes adipogenesis in 3T3-L1 cells by enhancing PPARγ2 and C/EBPα gene expression. Life Sci..

[29] Liu S., Wu Z., Guo S., Meng X., Chang X. (2018). Polyphenol-rich extract from wild Lonicera caerulea berry reduces cholesterol accumulation by mediating the expression of hepatic miR-33 and miR-122, HMGCR, and CYP7A1 in rats. J. Funct. Foods.

[30] Ha J., Shim Y.-S., Seo D., Kim K., Ito M., Nakagawa H. (2012). Determination of 22 Ginsenosides in Ginseng Products using Ultra-High-Performance Liquid Chromatography. J. Chromatogr. Sci..

[31] Townsend K.L., Tseng Y.-H. (2014). Brown fat fuel utilization and thermogenesis. Trends Endocrinol. Metab..

[32] Seale P., Conroe H.M., Estall J., Kajimura S., Frontini A., Ishibashi J., Cohen P., Cinti S., Spiegelman B.M. (2011). Prdm16 determines the thermogenic program of subcutaneous white adipose tissue in mice. J. Clin. Investig..

[33] Wang W., Seale P. (2016). Control of brown and beige fat development. Nat. Rev. Mol. Cell Biol..

[34] Ma X., Wang D., Zhao W., Xu L. (2018). Deciphering the Roles of PPARγ in Adipocytes via Dynamic Change of Transcription Complex. Front. Endocrinol..

[35] Mu Q., Fang X., Li X., Zhao D., Mo F., Jiang G., Yu N., Zhang Y., Guo Y., Fu M. (2015). Ginsenoside Rb1 promotes browning through regulation of PPARγ in 3T3-L1 adipocytes. Biochem. Biophys. Res. Commun..

[36] Giri S., Rattan R., Haq E., Khan M., Yasmin R., Won J.-S., Key L., Singh A.K., Singh I. (2006). AICAR inhibits adipocyte differentiation in 3T3L1 and restores metabolic alterations in diet-induced obesity mice model. Nutr. Metab..

[37] Hardie D.G., Ross F.A., Hawley S.A. (2012). AMPK: A nutrient and energy sensor that maintains energy homeostasis. Nat. Rev. Mol. Cell Biol..

[38] Wu L., Zhang L., Li B., Jiang H., Duan Y., Xie Z., Shuai L., Li J., Li J. (2018). AMP-Activated Protein Kinase (AMPK) Regulates Energy Metabolism through Modulating Thermogenesis in Adipose Tissue. Front. Physiol..

[39] Cantó C., Auwerx J. (2009). PGC-1alpha, SIRT1 and AMPK, an energy sensing network that controls energy expenditure. Curr. Opin. Lipidol..

[40] Lee J.E., Kang S.J., Choi S.H., Song C.H., Lee Y.J., Ku S.K. (2015). Fermentation of Green Tea with 2% Aquilariae lignum Increases the Anti-Diabetic Activity of Green Tea Aqueous Extracts in the High Fat-Fed Mouse. Nutrients.

